# Interpreting Immunoregulation in Lung Fibrosis: A New Branch of the Immune Model

**DOI:** 10.3389/fimmu.2021.690375

**Published:** 2021-08-20

**Authors:** François Huaux

**Affiliations:** Louvain Centre for Toxicology and Applied Pharmacology (LTAP), Institut de Recherche Experimentale et Clinique (IREC), Université Catholique de Louvain, Brussels, Belgium

**Keywords:** immunosuppression, inflammation, regulatory lymphocytes and myeloid cells, carbon nanotubes, silica and asbestos

## Abstract

Immunostimulation is recognized as an important contribution in lung fibrosis in some animal models and patient subsets. With this review, we illustrate an additional scenario covering the possible implication of immunoregulation during fibrogenesis. Available animal and human data indicate that pulmonary fibrosis also includes diverse and discrete immunoregulating populations comprising regulatory lymphocytes (T and B regs) and myeloid cells (immunosuppressive macrophages and myeloid-derived suppressive cells; MDSC). They are initially recruited to limit the establishment of deleterious inflammation but participate in the development of lung fibrosis by producing immunoregulatory mediators (mainly TGF-β1 and IL-10) that directly or indirectly stimulate fibroblasts and matrix protein deposition. The existence of this silent immunoregulatory environment sustains an alternative mechanism of fibrosis that explains why in some conditions neither pro-inflammatory cytokine deficiency nor steroid and immunosuppressive therapies limit lung fibrosis. Therefore, the persistent presence of immunoregulation is an important parameter to consider for refining therapeutical strategies in lung fibrotic disorders under non-immunostimulatory conditions.

## Diverse Lung Fibrotic Diseases With Diverse Mechanisms

Repair of damaged tissue is a fundamental biological process that allows the ordered replacement of dead or injured cells. However, although initially beneficial, the healing process becomes pathogenic when it is not controlled appropriately, leading to considerable tissue remodeling and the formation of permanent scar tissue and fibrosis (1). The major site of histopathological lung fibrosis is the interstitium, which consists of alveolar epithelium, pulmonary capillary endothelium, basement membrane, and perivascular and perilymphatic tissues. There are now almost 300 distinct injurious or inflammatory causes of interstitial lung disease that can result in progressive lung scarring. Many others are referred as idiopathic (i.e. idiopathic pulmonary fibrosis, or IPF) when it arises for no obvious reason (2). Silicosis is one the oldest recorded interstitial lung disease characterized by alveolitis and progressive nodular fibrosis (3). This fibroproliferative disorder can be traced back to ancient Egypt, where it was caused by inhalation of crystalline silica. It is also well known that long-term asbestos fiber inhalation causes asbestosis also comprising persistent nodular fibrosis (4). Although the incidence of silicosis and asbestosis has diminished, it continues to be a major cause of occupational lung disease in exposed workers, particularly in developing nations (5). More recently, carbon nanotubes (CNT), which present some of the physical characteristics of asbestos (long and rigid), also induce granulomatous lung disorder characterized by persistent immune responses, culminating in the development of lung fibrosis (6). Additionally, lung fibrogenic reaction may arise from other exogenous environmental stimuli such as organic dusts (bacterial, fungal and avian antigens) (7).

Suppression of chronic inflammation by immunosuppressive therapy turn off pulmonary fibrogenesis in some sub-groups of patients (e.g. non-specific interstitial pneumonia). These findings argue that inflammation represents a major pathological pathway in lung fibrosis (8, 9). A pathogenesis paradigm that did not require inflammation and immunostimulation was, however, proposed since there is little evidence of inflammation in the histopathological samples obtained from susceptible ageing individuals developing IPF undergoing surgical lung biopsy. Treatment with anti-inflammatory agents, such as steroids or anti-cytokines, seemed to have no effect on disease progression and outcome (10, 11). These last clinical observations strongly suggested that inflammation represents an important but dispensable event and that other mechanisms than immunostimulation exist or co-exist in pulmonary fibrosis (12, 13).

## The Relevance of Reliable Animal Models of Lung Fibrosis

It is unanimously recognized that animal models currently available are particularly useful to discover new pro-fibrotic mediators and pathological avenues related or unrelated to the inflammatory concept. The development of experimental models producing long-lasting lesions akin to those seen in human fibrosis and defined by progressive and irreversible matrix deposition has already been the subject of many studies (14). Presently, chemical insults such as those caused by bleomycin are widely used to induce fibrotic disease. Many research studies have focused on changes in inflammatory phenomena after a single instillation of bleomycin and have yielded similar findings: the lung injuries caused by bleomycin induce acute recruitment and activation of inflammatory leukocytes which produce mediators (cytokines, chemokines, growth factors, and prostaglandins) activating fibroblast and driving fibrotic disease. While bleomycin represents the preferred molecule in this context, the lung fibrosis obtained by an unique dose of this drug is not systematically progressive and resolves itself over time after bleomycin metabolization (15).

This discrepancy led researchers to explore other models and discover new pathological avenues not apparent in the acute bleomycin-induced fibrosis model, which mainly (if not exclusively) supports that inflammation drives fibrosis. Multiple instillation of bleomycin has previously been shown to recapitulate the epithelial remodeling and fibrosis progression in the lungs of patients with IPF (16). Indeed, a repetitive alveolar epithelial injury caused by repetitive intratracheal injections of bleomycin is now proposed as the initial and major event that triggers a series of repair pathways that are in some way aberrant, leading to inappropriate fibrosis. Repeated injuries prevent epithelial cell regeneration, re-epithelialization and epithelial structure restauration and lead to a sustained disruption of alveolar epithelial morphology and fibrogenesis in injured alveoli. This experimental approach thus represents an ultimate model to address mechanistic questions on epithelial remodeling (17).

Murine models of univocal chronic lung responses and progressive fibrotic lesions using inorganic particles (crystalline silica, asbestos and CNT) are well known to cover severe fibrotic respiratory disorders in exposed human. Instillation of mineral particles into mouse and rat lungs results in the development of fibrotic nodules that resemble lesions which develop in humans. Rodents exposed to silica particles present alveolar fibrotic nodules, increased pulmonary lymphoid tissue and enhanced numbers of macrophages in the broncho-alveolar lavage fluid. Interestingly, silica, asbestos and CNT are retained in the lung and the response is characterized by a persistent and progressive fibrosis (18). Based on findings on particle-induced pulmonary fibrosis in mice, it has been recently proposed a new hypothesis that the fibrotic response may result from an exaggerated and persistent immunoregulating responses instead of or along with inflammation. This new concept have been confirmed in human silicosis but also in other animal models and patients. These results, summarized hereafter, may explain how lung fibrosis can develop in absence of immunostimulation.

## History of Immunoregulating Surveillance

**Figure 1:**
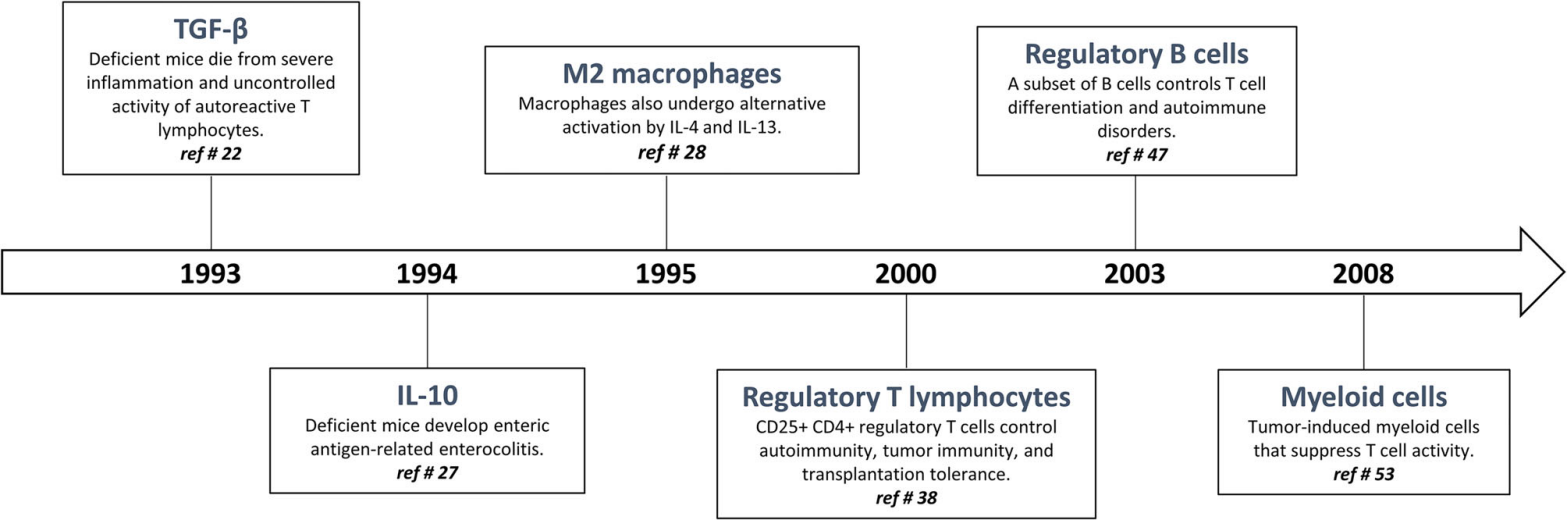
Timeline: historical progression of immunoregulation in the literature.

**Figure 2:**
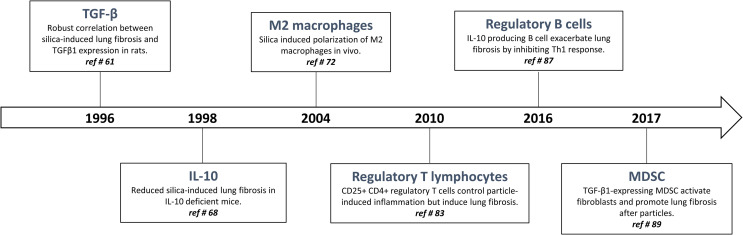
Timeline: historical progression of immunoregulation in particle-induced lung fibrosis.

The quality, magnitude, and persistence of immune reactions results from the balance between immunostimulating and immunoregulating responses. The basic mechanism of immunoregulation comprises an interconnecting system involving diverse anti-inflammatory and immunosuppressive cytokines, lymphocytes and myeloid cells. There is now evidence that this sophisticated immunoregulatory systems is crucial in maintaining immune homeostasis and resolving persistent inflammation. Immunoregulatory alterations are often implicated in the pathogenesis of several inflammatory diseases such as infection, allergy, and autoimmune disorders. Conversely, uncontrolled and exacerbated immunoregulation is strategically exploited by cancer cells to survive, proliferate and escape detection by anti-tumor effector T lymphocytes (19). A well-knowledge of immunoregulation now serves for future strategies of diagnosis and treatment. The following sections are devoted to describe (i) historical aspects of immunoregulatory mechanisms (**Figure 1**) and (ii) matching evidence supporting a deranged immunoregulation in pulmonary fibrogenesis (**Figure 2**).

### TGF-β1 and IL-10 as Master Immunoregulating Cytokines

The most striking and non-redundant function of TGF-β1 is to regulate the immunostimulatory and inflammatory responses by orchestrating immunoregulation (20, 21). Indeed, in 1993, Kulkarni and Karlsson demonstrated that mice deficient for the gene encoding TGF-β1 die rapidly from a multi-systemic inflammatory syndrome related to deleterious T cell autoreactivity (22) (**Figure 1**). To become active, mature TGF-β1 must be released from the LAP (latency-associated peptide), a process referred to as TGF-β1 activation (23). The best-characterized mechanism of TGF-β1 activation imply conformational changes in the latent TGF-β1 molecule involving interaction of latent TGF-β1 with integrin αVβ8 and GARP (glycoprotein A repeats predominant protein or LRRC32) (21). The binding of the active TGF-β1 peptide to its receptors leads to a series of signaling events that are mainly mediated by SMAD2/3/4 complex that binds SMAD response elements located in the promoter regions of many genes involved in the immunoregulatory responses (21).

The cytokine interleukin-10 (IL-10) was originally described as a ‘cytokine synthesis inhibitory factor’ (CSIF), which was produced by mouse T helper 2 (Th2) clones and inhibited cytokine production by activated Th1 clones (24). IL-10 reportedly inhibits nuclear factor-κB (NF-κB) activation and expression of the most inducible cytokines and chemokines that are involved in inflammation. The anti-inflammatory activities of IL-10 also include induction of IL-1 receptor antagonist (IL-1ra) (24) and soluble p55 and p75 TNF receptor production, indicating that IL-10 induces a shift from production of pro-inflammatory to anti-inflammatory mediators (25). IL-10 strongly inhibits cytokine production and proliferation of CD4+ effector T cells *via* its down-regulatory effects on APC function. IL-10 also induce the differentiation of naive Th cells in a subset of regulatory T cells which are defined by their ability to produce high levels of IL-10 and TGF-β1 (26). The IL-10-related potent immunosuppressive functions and its proximity to TGF-β1 are perfectly illustrated by the work of Kuhn and colleagues demonstrating that IL-10 deficient mice suffer from chronic enterocolitis characterized by extensive inflammatory reactions and aberrant expression of major histocompatibility complex class II molecules (27) (**Figure 1**).

### M2/Immunoregulatory Macrophages and Type 2 Like Immune Response

During the 1990s, Gordon and colleagues demonstrated that IL-4 and IL-13 induced an “alternative” form of activation in macrophages beside the “classically” polarized macrophages (28, 29) (**Figure 1**). In 2000, Mills and colleagues identified the fundamental M1/M2 polarization axis of macrophages and proposed that M1/inflammatory macrophages are necessary to eliminate infectious organisms and tumor cells while M2/immunoregulatory macrophages are implicated in repair process and wounds. Importantly, the uncontrolled M1 and M2 activation is associated with tissue injury and cancer progression, respectively (30, 31).

The so-called M2 phenotype express anti-inflammatory and immunosuppressive mediators such as IL-10 and TGF-β1, specific chemokines (CCL17 and CCL22, C-C motif), the subclass B scavenger receptor (CD 163), and the mannose C receptor type 1 (CD206 or MRC1) (32). The TGF-β1- and IL-10 producing M2 macrophages are thus intimately involved in the regulation of immune responses. Transcription factors such as IRF4 and STAT6 are required for M2 macrophage activation and differentiation. These different stimuli are likely to initiate during Th2-like responses (33). Th2 responses and cytokines (mainly IL-4 and IL-13) induce the differentiation of M2 macrophages.

The model categorizing macrophages (or T helper cells) in only two divergent groups M1 vs M2 (or Th1 and Th2) does not cover the intrinsic heterogeneity of macrophages (and T lymphocytes) now identified and described in diseases. A consensus now refers to M1-like or M2 like-subpopulations for avoiding oversimplified categories and highlighting the diversity and versatility of macrophages (34). M2-like macrophages possessing various functions, characteristics and deleterious activities are well known to be crucial in asthma, interstitial lung diseases and cancer (35, 36).

### Regulatory T Cells

Regulatory T cells were initially described by Gershon et al. in the early 1970s and were called suppressive T cells because they practiced immune suppression (37). Unfortunately, despite the importance of these studies there was extensive skepticism in the immunological field about the existence of these cells, and suppressive T cells left the centre stage of immunology for decades. However, in 1996, Sakaguchi and colleagues rehabilitated the concept of “suppressive T cells”, now renamed “regulatory T cells” (38) (**Figure 1**). CD4+ Foxp3+ regulatory T cells (T regs) constitute a thymus-derived sub-population of CD4+ T lymphocytes that constitutively express the transcription factor Foxp3 (forkhead box P3), required for their development and their anti-inflammatory and immunosuppressive function (39, 40). T regs are developed in the thymus (naturally occurring T regs; nT regs) or are differentiated from naive T cells in the presence of TGF-β1 following T-cell receptor stimulation (induced T regs; iT regs) (41). These lymphocytes are crucial to maintain tolerance by downregulating undesired immune responses to self and non-self-antigens (42, 43). Absence or defective function of regulatory T cells is correlated with the development of immuno-pathologies such as auto-immune diseases (e.g. psoriasis and rheumatoid arthritis) and asthma. In contrast, their accumulation is associated with tolerance and implicated, for instance, in the development of cancer (44). Several mechanisms have been proposed to explain immunosuppressive functions of T regs. These include secretion by T regs of immunosuppressive cytokines, cell-contact-dependent suppression and functional modulation. Most *in vivo* studies indicate that T regs mediate immunosuppression by producing IL-10 and TGF-β1 (45). The strong immunosuppressive activity T regs is also related to their capacity to regulate the polarization and function of effector T lymphocytes (T eff, i.e. Th1, Th2, and Th17).

### B Regs

The historical description of the new B-cell subtype named regulatory B-cells (B regs) dates back to 1974. In a mouse model of eczema, adoptive transfer of total splenocytes had a suppressive effect, whereas adoptive transfer of splenocytes from which B-lymphocytes were removed had no effect (46). In 2003, the regulatory role of B cells was shown in a mouse model of experimental autoimmune disease and this regulatory role was attributed to the ability of B cells to produce IL-10 (47) (**Figure 1**). B cells have been shown to restore homeostasis, possess important immunosuppressive functions and play a key role in disease control and immune tolerance (48, 49). IL‐10‐producing B cells produce anti‐inflammatory IgG4 and activate Treg cells. By promoting M2-like macrophage polarization, B regs cells also reduce auto‐reactive Th1 and Th17 cell responses and limit damaging inflammatory responses. In contrast, B regs impair cytotoxic T‐cell and NK cell responses to tumor cells and thereby promote progression of cancer (50). Beside IL-10, the exact mechanism by which B regs act *in vivo* remains unclear. A close contact between B-lymphocytes and the T lymphocytes, notably through the CD40-CD40L pathway are required (51, 52).

### Myeloid Derived Suppressive Cells

MDSC were first identified by Van Ginderachter and his team in 2008. They detected a discrete myeloid-derived suppressor cell subpopulations with T cell-suppressive activity invading tumors. Thus, it was suggested that this MDSC population is responsible for down-regulating immune responses related to tumor progression and metastasis (53) (**Figure 1**). MDSC are now defined as heterogeneous and immature myeloid cells generated from the bone marrow and active in cancer development and inflammation regulation (54). Under normal physiological conditions, MDSC are rapidly differentiated into mature granulocytes, macrophages and dendritic cells. However, the differentiation of these cells into mature myeloid cells is blocked in chronic pathologies such as cancer (54). Two categories of these cells can be observed, one with granulocyte morphology and the other with monocyte morphology (55). MDSC suppress CD8, CD4 T cell activities and NK cell activity by inducing a suppressive environment (56). Several different mechanisms of action have been identified to explain the strong immunosuppressive activity of MDSC involving direct cell contacts and/or the production of several released mediators (54). MDSCs have the ability to induce differentiation and expansion of regulatory T cells by producing cytokines such as IL-10 and TGF-β1 or *via* CD40 (57). These cells can also deprive T cells of amino acids essential for their activity such as arginine, which is necessary for lymphocyte activation. Arginase, highly expressed by MDSCs, metabolizes arginine to urea and ornithine. Indoleamine-2.3-dioxygenase (IDO), an enzyme that catalyzes tryptophan, an essential amino acid for T cells, might also be involved in the immunosuppressive mechanisms of MDSC (54).

## The Discovery of Immunoregulation in Particle-Induced Lung Fibrosis

Inorganic particle-induced lung fibrosis consists of an uncontrolled inflammation of the respiratory system tissues characterized by a chronic macrophage and neutrophil infiltration that ultimately causes fibrosis (58). Based on the widespread study of Piguet and colleagues published in 1990 in Nature, it is accepted that silica particles activate intracellular signaling pathways that culminate in the production of the pro-inflammatory mediator TNF-α, which is crucial in driving alveolitis and lung fibrosis (59). Additionally, a recent and pivotal study demonstrated that silica activate caspase-1 in a NALP3 inflammasome-dependent manner leading to the processing and secretion of the pro-inflammatory cytokine IL-1β. Evidence demonstrate that IL-1β initiates a cascade of reactions leading to inflammation and uncontrolled fibrosis (60). These observations support the concept that inflammation and sustained expression of inflammatory cell-secreted pro-fibrotic cytokines participate in the etiology of fibroproliferative diseases associated to inorganic particles.

The interconnection between inflammation and fibrosis was, however, questioned when active TGF-β1 was pointed as a key pro-fibrotic factor in response to particles. In silicosis and asbestosis, TGF-β1 expression has been found to be increased in lung tissues from patients with accelerated fibrotic disease progression (7) (**Figure 2**). The crucial activity of TGF-β1 during fibrogenesis has been unanimously recognized in the experimental studies using silica-, asbestos- and CNT-treated mice (6, 61–63). TGF-β1 is a major profibrogenic cytokine by delaying epithelial wound healing and expanding mesenchymal compartment (64). While the activity of TGF-β1 on fibroblasts is undisputed and not anymore debated, these observations intrigued immunologists because the presence of this powerful immunosuppressive cytokine (see above) was not in line with the IL-1/TNF-related immunostimulation axis.

Additional data challenged the inflammatory concept. IL-10, another potent immunoregulatory cytokine, has also fibrogenic activities in responses to fibrogenic particles. To examine the immune responses over the whole course of the pathological process induced by fibers and particles, validated experimental models in mice and rats with contrasting sensitivities were developed (65). By comparing these models and using deficient mice, it was newly discovered that IL-10 produced by particle-activated macrophages limit neutrophilic inflammation but is a key mediator implicated in the fibrotic lung response to silica by controlling the balance between pro- and anti-fibrotic mediator production (respectively TGF-β1 and prostaglandin E2) (65–68) (**Figure 2**). IL-10 is also a prevailing inducer of type-2 immune responses in particular M2-like and Th2-like pro-fibrotic cells (69, 70). The fibrogenic activity of macrophage-derived IL-10 and M2-like macrophages is not limited to silicosis (71, 72). Indeed, these macrophages are also implicated in the fibrotic lung response induced by asbestos and CNT (6, 73, 74) (**Figure 2**).

The little evidence of inflammation in the histopathological samples obtained from silicotic patients undergoing surgical lung biopsy (75) consolidate the pathogenesis paradigm that did not require inflammation. In contrast, high levels of IL-10 in BAL or serum were detected in patients with silicosis and asbestosis in the absence of clear inflammatory reaction (73, 76). Treatment with anti-inflammatory agents, such as corticosteroids, seemed to have no effect on outcome of these fibrotic diseases (75, 77, 78). These human observation are in accordance with several animal data indicating that steroid and numerous anti-inflammatory strategies reduce particle-induced lung inflammation but not IL-10-TGF-β1 expression and lung fibrosis in sensitive animals (79, 80). At this time, it was thus suggested that these two immunoregulatory cytokines are the major event that triggers a series of repair pathways that are aberrant and lead to inappropriate fibrosis under non-inflammatory conditions.

According to the concept that inflammation is responsible for fibrogenesis and given their ability to dampen inflammatory responses, CD4+ regulatory T lymphocytes (T regs) were first supposed to slow the progression of fibrosis (81). Indeed, it is well recognized that T regs, by restraining effector T cell responses and inducing tolerance through the production of TGF-β1 and/or IL-10, control lung inflammatory disorders (82). However, CD4+ Foxp3+ regulatory T cells are persistently recruited during long-term responses to particles (69, 83) (**Figure 2**). T regs purified from the lung of silica-treated mice highly express fibrogenic mediators, stimulate fibroblast proliferation *in vitro* and increase lung collagen deposition upon transfer into naive mice. Interestingly, the effects of T regs on fibroblast proliferation recapitulate the main function of PDGF as a primary mitogen for fibroblasts during lung fibrosis. The stimulatory effect of T regs on fibroblasts *in vitro* and *in vivo* was completely abolished by a PDGF receptor inhibitor (imatinib mesylate). It is thus likely that the role of T regs is to increase tissue fibroblast numbers, and consequently, amplify the subsequent fibroblast activation and collagen deposition (83). The pro-fibrogenic functions of T regs also comprise a stimulatory activity on Th2-like pro-fibrotic cells (69). In contrast to what it is thought, T regs thus participate in the fibrogenesis and are able to aggravate lung fibrosis induced by fibrogenic particles (i.e. silica and asbestos) in absence of inflammation (74). Finally, Xin and colleagues also observed a clear balance between inflammatory Th effector cells (Th1 and Th17) and T regs in mice treated with silica (84, 85). Neutralization of T regs-immunosuppressive activity resulted in enhanced lung inflammation and Th17 accumulation further demonstrating that T regs are initially recruited to control inflammatory responses (85, 86).

Based on the immunosuppressive profile of silica-treated mice, other immunosuppressive populations among lymphocytes have been investigated. Regulatory B lymphocytes (B regs), another immunosuppressive cell population (see above), also accumulate and participate in granuloma formation and fibrosis development by producing lung IL-10 in mice treated with silica. A heightened accumulation of inflammatory T effector cells (Th1 and Th17) but limited pulmonary fibrosis were observed in B reg-depleted mice treated with silica (87) (**Figure 2**). IL-10-producing B regs were also noted in silicotic patients in absence of inflammatory reaction (76). B regs exacerbate fibrogenesis by stimulating T regs functions and polarization *via* the release of IL-10 (88). These findings indicated that the accumulation and polarization of immunoregulatory lymphocytes is a central event during particle-induced pulmonary fibrosis with limited immunostimulation.

Finally, M-MDSC are also progressively and specifically accumulated during the development of pulmonary fibrosis. Indeed, a close relationship between the accumulation of MDSC, pulmonary immunosuppression and lung fibrosis was clearly found in mice treated with silica or CNT (89, 90) (**Figure 2**). Beside M2-like macrophages and T regs, immunosuppressive MDSC also expressed TGF-β1 conferring to these myeloid cells the capacity to down regulate T effector cell activity (91). In order to define their role in fibrosis, lung MDSC were purified from silica-treated mice and co-cultured with naive lung fibroblasts. MDSC stimulates lung fibroblasts to release tissue inhibitor of metalloproteinase and collagenolytic activity by expressing TGF-β1. They contribute to lung fibrogenesis by inducing a non-degrading collagen microenvironment (89).

The persistent accumulation of immunoregulatory macrophages, lymphocytes and myeloid cells in the lung during the progressive establishment of experimental silicosis is consistent with studies on tuberculosis (92) and lung cancer (93) that often affect the silicotic patients. Indeed, these cells control neutrophilic inflammation and anti-tumor T effector lymphocytes dedicated to microorganism and tumoral cell elimination. However, authors found that human silicosis is accompanied by a reduced number of blood regulatory T cells and speculated that the absence of these regulatory cells may explain the occurrence of autoimmune diseases (e.g. systemic scleroderma, rheumatoid arthritis and systemic lupus erythematosus) (94, 95). These conflicting results highlight the possible limitations of the mouse models. Injection of silica in mice does not fulfill all conditions encountered in patients with silicosis (infection, cancer and autoimmune diseases). Moreover, experimental models used to study the effects of silica are relatively short compared to human silicosis. These contradictory results also suggest that the fibrogenic activity of immunoregulation is only effective in a non-inflammatory environment.

## Inclusive Implication of Immunoregulatory Mediators And Cells in Lung Fibrotic Diseases

Immunoregulation is operative in different fibrotic context and not specifically concomitant to particle-induced fibrogenesis. Indeed, recent investigations using complementary mouse models of lung fibrosis also reported that inflammation is not an absolute prerequisite for fibrogenesis and that the fibrotic pathological process can develop through immunoregulation.

IL-10 induce lung collagen deposition and fibrosis when overexpressed in transgenic mice (96, 97). These observations correspond to those noted when TGF-β1 is overexpressed in murine lungs (98). Intratracheal transfer of adenoviral recombinant IL-10 or TGF-β1 to murine lung has been shown to dramatically increase fibroblast accumulation and expression of type I and type III collagen around airways as well as in the pulmonary interstitium (70, 99, 100). The pro-fibrotic function of IL-10 is associated to infiltration of fibrocytes and M2-like macrophages (97). In addition; IL-10 and TGF-β1 contribute to lung injury and fibrosis by sensitizing epithelial cells to apoptosis (98, 101). TIM-3+ M2-like macrophages that possess strong immunoregulatory functions is now considered as an important pro-fibrotic population by being a key source of TGF-β1 and IL-10. Adoptive transfer of this immunoregulatory population promoted bleomycin-induced lung fibrosis by highly secreting TGF-β1 and IL-10 (102). Recent data at single cell level suggest that pro-fibrotic macrophages did not demonstrate a shaped and clear M2-like polarization but resemble to alveolar macrophages deriving from monocytes (103). Altogether, these studies indicated that the long-term overexpression of M2 like-related immunoregulating cytokines that suppress inflammation such as TGF-β1 and IL-10 are also profibrotic factors in the lung.

*In vivo* expansion of lung CD4+CD25+Foxp3+ T regs cells during bleomycin-induced lung fibrosis unexpectedly led to an increase of fibrogenesis. More important, this pro-fibrotic effect was a lymphocyte-dependent process. A marked down-regulation of type 1 and an increase of type 2 immune responses in the lungs were proposed to explain T reg fibrogenic activity (104, 105). These observations were corroborated by Chakraborthy and colleagues. Depletion of T regs ameliorate bleomycin-induced acute lung fibrosis by modulating Th effector cell balance. In addition, adoptive transfer of Sema 7a1 T regs induces fibrosis in the TGF-β1–exposed murine lung by altering the production of T-cell mediators (106).

**Figure 3:**
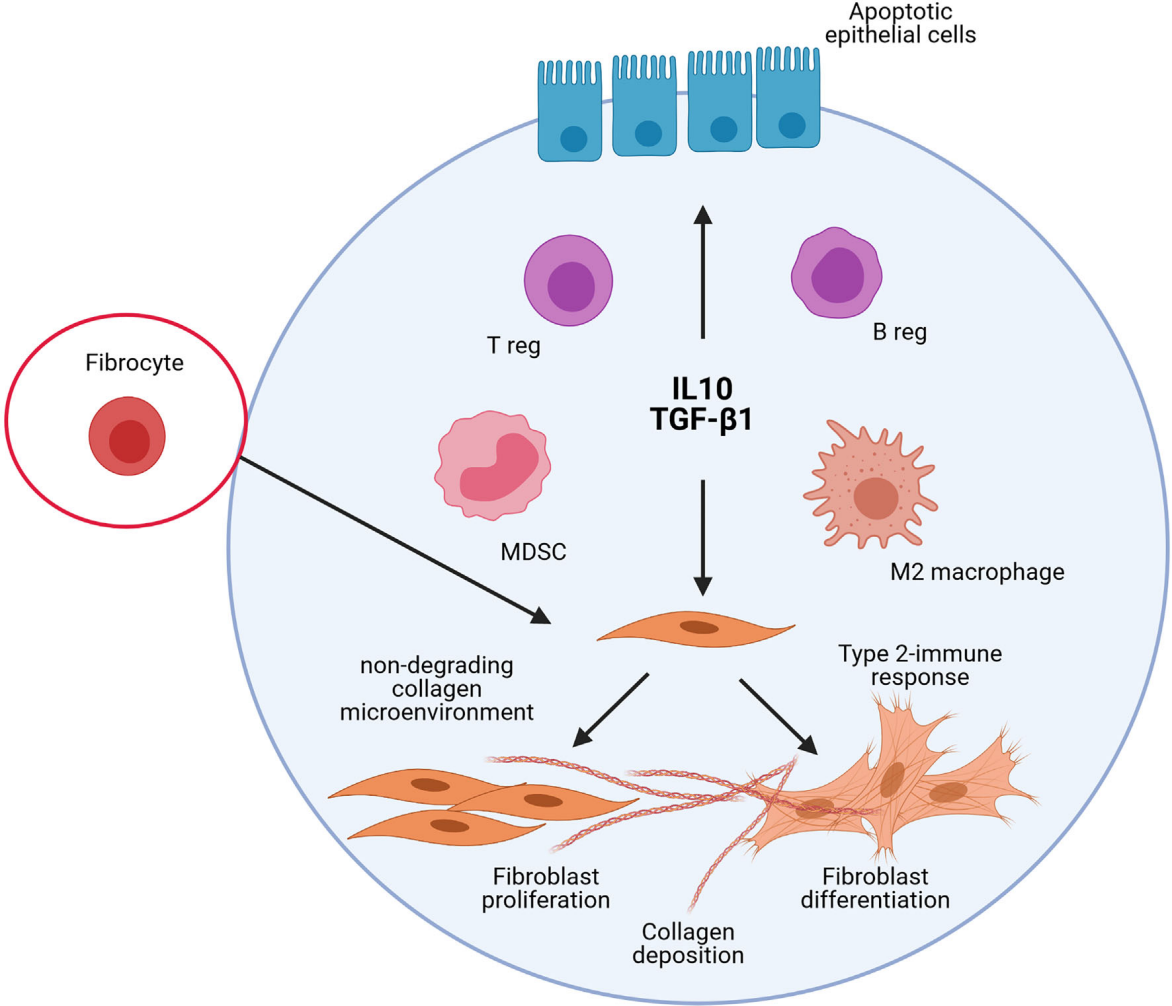
Schematic representation of the implication of the different immunoregulatory cells and mediators in lung fibrosis. The studies on experimental lung fibrosis have highlighted a new pathological pathway, which suggests that pulmonary fibrosis is orchestrated by an immunoregulatory response characterized by a persistent accumulation of pulmonary immunoregulatory cells (regulatory T and B lymphocytes, i.e. T and B regs; regulatory myeloid cells, i.e. M2/immunosuppressive macrophages and Myeloid Derived Suppressive Cells, MDSC) and a sustained production of IL-10 and TGF-β1. The persistent accumulation of these elements to control immunostimulatory responses in the lungs contribute, however, to pulmonary fibrosis. This sustains the view that immunoregulation is important pro-fibrotic environment that could markedly explain the development of the lung fibrotic response under non-inflammatory conditions.

Accumulation of MDSC with functional immunosuppressive activity was also noted in bleomycin-induced experimental pulmonary fibrosis and their potential role in fibroblast activation investigated (107, 108). Purified MDSC differentiate into lung fibroblasts as manifested by significantly elevated α-smooth muscle actin and TGF-β1 expression. Differentiation of MDSC into fibrocytes could also be possible during tissue repair processes. Indeed, there is some evidence that peripheral monocytes and MDSCs differentiate into fibrocytes (109, 110). These cells have the ability to promote fibroblast proliferation, migration, and collagen production but also differentiate into myofibroblasts (111, 112). Altogether, these previous experimental studies are well in accordance with the immunoregulation concept elaborated from findings reported after particle exposure and supporting that persitent immunoregulatory environment is profibrotic in absence of immunostimulation (**Figure 3**).

The disconnection between fibrosis and inflammation is not limited to patients developing silicosis or asbestosis. Clinical measurements of inflammation in IPF patients developing fibrosis fail to correlate scar formation with inflammation and immunostimulation. Corticosteroids have never conclusively been shown to significantly alter the course of pulmonary fibrosis in patients and have, at best, limited efficacy in the treatment of scarring disease (113).

Interestingly, the expression TGF-β1 and IL-10 mainly by macrophages was increased in lung biopsies from patients with IPF compared with controls, suggesting the presence of M2-like macrophages (114, 115). Single-cell multi-omics approaches characterizing macrophage populations in health and lung fibrotic disease at high resolution tempered a clear accumulation of M2-like polarized macrophages in fibrotic tissue. Fibrotic macrophages in IPF patients are now identified as proliferating SSP1-positive macrophages (116, 117). However, it has been suggested that Th2-related cytokines such IL-4 and IL-3 as well as M-CSF activate this subset of proliferating macrophages (117). Clinical reports showed increased number and function of CD4+CD25+FoxP3+ T regs in the lungs and blood of patients with IPF associated with a more progressive clinical course (106, 116, 118–120). More recently, the importance of MDSC was also suggested by the observation of MDSC accumulation in IPF lungs (108, 121).

In conclusion, the presence of immunoregulatory microenvironment may be relevant to human pathology. Based on these recent findings, it is important to consider the possibility that regulatory lymphocytes and myeloid cells may also drive fibroproliferative wound healing. Consequently, these cells and their cytokine products could become therapeutic targets in patients developing fibrotic diseases. Particularly, these studies identified as potentially important targets the production of TGF-β1 and IL-10 by immunoregulatory cells in non-immunostimulatory conditions. The clinical separation of patients reaches from immunoregulatory or immunostimulatory scar formation could offer novel markers for the pathological assessment as well as novel regulators and drug targets to treat pulmonary fibroproliferative diseases.

## Author Contributions

The author confirms being the sole contributor of this work and has approved it for publication.

## Funding

This work was funded by the Actions de Recherche Concertées, Fédération Wallonie-Bruxelles (ARC 19/24-098, CYTAID), Fondation Contre le Cancer (2019-219), Fonds de la Recherche Scientifique (FNRS, PDR T.0119.13), ANSES (Agence nationale française de sécurité sanitaire de l’alimentation, de l’environnement et du travail, MacFibOsis) and European Commission under H2020 project (Contract no. 874707, Eximious). FH is a Senior Research Associate with the FNRS, Belgium.

## Conflict of Interest

The author declares that the research was conducted in the absence of any commercial or financial relationships that could be construed as a potential conflict of interest.

## Publisher’s Note

All claims expressed in this article are solely those of the authors and do not necessarily represent those of their affiliated organizations, or those of the publisher, the editors and the reviewers. Any product that may be evaluated in this article, or claim that may be made by its manufacturer, is not guaranteed or endorsed by the publisher.
